# Engineered bacteriophytochrome heterodimers for research and applications

**DOI:** 10.1016/j.jbc.2025.110452

**Published:** 2025-07-04

**Authors:** Iida Tuure, Cornelia Böhm, Jessica Rumfeldt, Elina Multamäki, Heikki Takala

**Affiliations:** 1 Department of Biological and Environmental Science, Nanoscience Center, University of Jyvaskyla, Jyvaskyla, Finland; 2 Department of Anatomy, University of Helsinki, Helsinki, Finland

**Keywords:** protein heterodimers, histidine kinase, phytochrome, photoreceptor, dimerization, optogenetics, protein kinase, protein phosphatase

## Abstract

Many proteins are dimeric, functioning as complexes of two identical or different subunits. Bacteriophytochromes are homodimeric photoreceptor proteins that sense red/far-red light with a photosensory module (PSM) and convert it to a biological response *via* an output module, usually a histidine kinase (HK). Here, we generate monomeric bacteriophytochrome PSMs that form stable heterodimers once mixed by modifying two salt bridges at the dimerization interface of the *Deinococcus radiodurans* phytochrome (*Dr*BphP). We confirm that these heterodimeric PSMs can control output HK module activity in response to red light and reveal that dimerization is required for kinase activity of the model HK FixL, but not necessarily for phosphatase activity of *Dr*BphP. By applying the heterodimeric variants to a red light-regulated gene expression tool, we exemplify the combined control of cellular events using both heterodimerization and light. These results pave the way for new heterodimeric systems, for example, in receptor protein research and optogenetics.

Proteins are key components of biological systems, and protein–protein interactions are crucial for a myriad of cellular processes such as metabolic pathways, signaling cascades, gene regulation, and enzymatic reactions. The most common oligomeric state for proteins is a dimer, a complex of either two identical (homodimer) or two different (heterodimer) subunits (1). The dimeric form is advantageous for several reasons: It takes less genetic material to encode a dimeric protein than a monomeric protein with similar molecular weight, thus reducing the probability of errors during DNA replication or translation as well as translation time. Dimerization also makes protein folding energetically more favorable and increases thermal stability compared to monomeric proteins (1). Additionally, the catalytic efficiency of enzymes is often reduced or lost in the monomeric forms of dimeric enzymes. Homodimers are three times more abundant in nature than heterodimers (1). This is likely because formation of homodimers is genetically more favorable, and proteins statistically tend to self-dimerize more strongly than to interact with other proteins (2). However, heterodimeric interactions may offer new functionalities over homodimers, and these are commonly present in the function and inhibition of enzymes, regulators and immune protein complexes (3). Studying interprotomer communication is important for understanding the function of dimeric proteins, as it is crucial for drug design and biotechnology. For homodimeric proteins, only symmetrical modifications are applicable, which limits the studies of the protomer–protomer co-operativity. This challenge can be overcome by artificial heterodimers.

Here, we engineered stable red light-controllable heterodimeric modules from a homodimeric phytochrome. Phytochromes are dimeric photoreceptor proteins that perceive the red and far-red region of the visible light spectrum. In addition to bacteria, they are encountered in plants, fungi and algae. Bacteriophytochromes (BphPs) are typically homodimers that comprise a conserved N-terminal photosensory module (PSM) which is followed by a C-terminal output module (OPM) (Fig. 1*A*) (4). The PSM is further divided into PAS (period/ARNT/single-minded), GAF (GMP phosphodiesterase/adenylyl cyclase/FhlA), and PHY (phytochrome-specific) domains. Within the GAF domain lies the red-light sensing chromophore, biliverdin IXα (BV), which is covalently linked to a conserved cysteine residue preceding the PAS domain core (5, 6). BphPs switch between two structurally and functionally different red light absorbing (Pr) and far-red light absorbing (Pfr) states that feature a characteristic Q-band absorption maximum, at 700 nm or 750 nm, respectively (Fig. 1*D*). *Dr*BphP, the model BphP from *Deinococcus radiodurans* also studied here, adopts Pr as its resting state, to which it thermally reverts over time in darkness (7).

**Figure 1:**
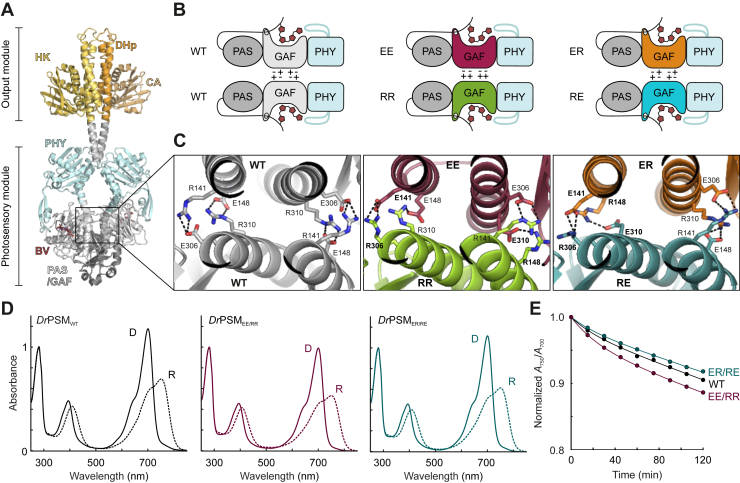
**Rational design of phytochrome heterodimer variants**. *A*, structural model of the full-length *Dr*BphP dimer. The structural presentation is derived from the cryogenic electron microscopy model of *Dr*BphP (pdb 8AVW) (8), combined with a histidine kinase module (HK) arrangement derived from *Thermotoga maritima* (pdb 2C2A) (74). The HK module is further divided into a dimerization and histidine phosphotransfer (DHp) domain and a catalytic ATP-binding (CA) domain. *B*, schematic illustration of phytochrome fragments used. The salt bridge-forming R and E residues are denoted as plus (+) and minus (−) signs, respectively. *C*, AlphaFold models of the GAF-GAF interface showing the four salt bridges in the wild-type *Dr*BphP (WT, *grey*) as well as the modified EE/RR heterodimer (*purple/green*) and ER/RE heterodimer (*orange/cyan*) pairs. Mutated amino acids are marked as *bold letters*. *D*, absorption spectra of wild-type (WT) *Dr*PSM as well as the EE/RR and ER/RE heterodimers in their dark-adapted (D) and red light illuminated (R) states. *E*, dark reversion of the three variants plotted as a change in *A*_750_/*A*_700_ over time. The reversions are normalized to the *A*_750_/*A*_700_ ratio at zero timepoint, *i.e.*, to the ratio of the red illuminated spectra.

The light activation of BphPs arises from isomerization of BV, which induces conformational changes in the PSM (8, 9, 10, 11). These conformational changes are relayed to the OPM, which is a histidine kinase (HK) module in *Dr*BphP and many other BphPs (4, 8, 12). The HK module contains a catalytic ATP-binding (CA) domain that catalyzes the transient phosphorylation of a conserved histidine residue at the dimerization and histidine phosphotransfer (DHp) domain (Fig. 1*A*). The BphPs that contain such a HK module commonly act as dimeric sensor histidine kinases (SHKs) in bacterial two-component signaling systems. In response to an incident signal, an SHK (de)phosphorylates its cognate response regulator (RR) protein that usually functions as a transcription activator (13, 14, 15). In the case of *Dr*BphP, a red light signal induces phosphatase activity, leading to dephosphorylation of the cognate response regulator *Dr*RR (12).

To provide tools for controlled dimerization and apply them to studies of SHK signal integration, we generated stable heterodimeric modules from the PSM of *Dr*BphP (*Dr*PSM) by introducing rationally selected salt bridge inversions into the *Dr*PSM dimerization interface (Fig. 1*B*). The resulting heterodimer PSM variants do not self-associate in isolation, but once mixed, they dimerize with their cognate heterodimer pair while retaining function with regard to light perception and signal integration. We have previously shown that an isolated protomer of *Dr*PSM undergoes similar structural changes as within the PSM dimer (16), but information on how this change is relayed between the sister protomers and further to the output HK module remains incomplete. Here we demonstrate that our heterodimeric *Dr*PSM variants can be used to study interprotomer communication within *Dr*BphP and other SHKs in a controlled fashion. Our results reveal that the kinase activity of one model SHK (FixL) requires PSM dimerization, but the phosphatase activity of another (*Dr*BphP) does not.

Further applicability of the heterodimeric *Dr*PSMs lies within the field of optogenetics, which aims to control cellular events with light by re-engineering natural photoreceptors (17). BphPs have several advantages over other photoreceptors in optogenetics: They respond to red/far-red light that carries less energy and penetrates tissues better than blue light, they are soluble and switchable between two metastable activity states, and they use a BV chromophore which is natively found in mammalian tissues (18, 19, 20). BphP PSMs have been successfully used to control phosphodiesterases (21, 22), guanylate/adenylate cyclases (23, 24), di-guanylate cyclases (25, 26), and tyrosine kinases (27, 28). Heterodimeric optogenetic tools up to date rely on light-controlled formation of transient heterodimers, either between two photoreceptors (29) or between a photoreceptor and an interacting protein (30, 31), whilst stable heterodimer tools are still lacking. Using the optogenetic pREDusk tool (32), we show here that the heterodimeric *Dr*PSM modules also remain controllable in bacterial cells. These results showcase that our heterodimeric *Dr*PSM variants provide an unprecedented combination of control over directed stable dimer formation and red-light control of photoreceptor activity, which paves the way for new kinds of optogenetic applications.

## Results

### Rational engineering of heterodimer variants

*Dr*BphP contains two dimerization interfaces, one between the GAF domains in the PSM and another between the HK OPMs (33, 34) (Fig. 1*A*). The GAF–GAF interface has previously been modified with three point mutations (F145S, L311E, and L314E) to create a monomeric *Dr*BphP variant (16, 33, 34). Taking this into account, we examined the surrounding residues for possible mutations that would allow the creation of heterodimers. According to dimeric *Dr*BphP crystal structures (5, 11, 35), the mainly hydrophobic dimerization interface between the GAF domains contains four salt bridges formed between two arginine (R141 and R310) and two glutamic acid residues (E148 and E306). We utilized these interactions and swapped the residues around to create four distinct variants: R141E/R310E, E148R/E306R, R141E/E148R, and E306R/R310E, referred to in this study as EE, RR, ER, and RE, respectively (Fig. 1, *B* and *C*). These mutations are designed to allow EE/RR as well as ER/RE heterodimerization through salt bridge formation but to prevent homodimerization by inducing repulsive forces at the GAF-GAF interface. As controls, we included the known monomerizing *Dr*PSM mutations F145S, L311E, and L314E (33, 34), resulting in monomeric variant *Dr*PSM_mon_. Wild-type *Dr*PSM (*Dr*PSM_WT_) is considered here as the stable dimeric control. For a full list of variants used in this study and their abbreviations, see Fig. S1*B*.

### Characterization of controlled PSM heterodimerization

UV-vis absorption spectroscopy was applied to study the effects of our heterodimer mutations on the BphP photocycle. The spectra of the heterodimer mixtures (*Dr*PSM_EE/RR_ and *Dr*PSM_ER/RE_) and the individual variants resembled those of prototypical BphPs and *Dr*PSM_WT_, with characteristic Soret (400 nm) and Q-band (700 nm) absorption peaks for both Pr and Pfr states (Figs. 1*D* and S2*A*). The dark reversion of the heterodimer mixtures also resembled that of *Dr*PSM_WT_ (Fig. 1*E*), whereas the individual components did not (Fig. S2*B*): *Dr*PSM_RR_ and *Dr*PSM_RE_ had slightly faster reversion, whereas *Dr*PSM_EE_ and *Dr*PSM_ER_ resembled the monomeric *Dr*PSM_mon_ variant by reverting very slowly (34). The lack of GAF-GAF interactions in *Dr*BphP_mon_ has been proposed to completely suppress dark reversion (34); however, our results imply that the reversion kinetics are affected not only by the monomerization of the variants but also by the point mutations themselves.

**Figure 2:**
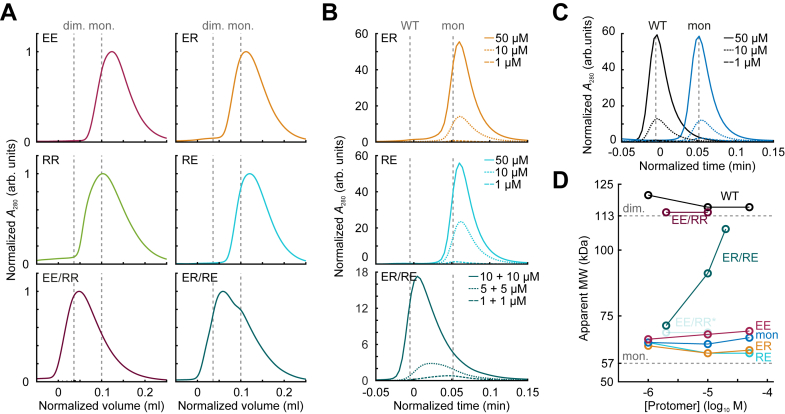
**Dark reversion and oligomeric state of the heterodimer variants.***A*, fast protein liquid chromatography (FPLC) of individual heterodimer variants and their mixtures. Theoretical retention positions of dimeric (113 kDa, “dim.”) and monomeric *Dr*PSM (57 kDa, “mon.”), calculated with the ProtParam tool (75) by using the *Dr*PSM sequence, are indicated as vertical lines for comparison. Peaks are normalized to the maximum of each peak. *B*, high-performance liquid chromatography (HPLC) of *Dr*PSM_ER_, *Dr*PSM_RE_ and their mixture at different concentrations. See Fig. S3*A* for *Dr*PSM_EE_, *Dr*PSM_RR_ and their mixture. Vertical lines indicate the elution of the dimeric *Dr*PSM_WT_ (“WT”) and monomeric *Dr*PSM_mon_ (“mon”) controls shown in panel *C* and in Fig. S3*B*. The chromatograms are normalized to the highest peak of 1 μM *Dr*PSM_WT_. See Fig. S3 for all variants and their retentions after *red* illumination. *C*, HPLC of dimeric *Dr*PSM_WT_ (“WT”) and monomeric *Dr*PSM_mon_ (“mon”) control at different concentrations. *D*, overview of the apparent molecular weights of the heterodimeric *Dr*PSM variants and their mixtures in dark plotted against increasing protomer concentration. The apparent molecular weights were derived from the HPLC measurements (*panel B* and *C*, and Fig. S3) and theoretical molecular weight (MW) positions of dimeric (dim.) and monomeric (mon.) *Dr*PSM are indicated as horizontal lines. The apparent monomeric fraction in the EE/RR mixture (∗) is likely caused by RR instability and resulting excess EE protomers in the mixture. For standard chromatograms and calibration curves, see Fig S3, *D* and *E*.

To verify the oligomeric state of the heterodimer variants, we applied fast protein liquid chromatography (FPLC) analysis to the heterodimer variants, either by themselves or as mixtures (Fig. 2*A*). The individual variants (*Dr*PSM_EE_, *Dr*PSM_RR_, *Dr*PSM_ER_, and *Dr*PSM_RE_) eluted corresponding to the size of a theoretical *Dr*PSM monomer, whereas the mixtures (*Dr*PSM_EE/RR_ and *Dr*PSM_ER/RE_) eluted resembling the size of a theoretical *Dr*PSM dimer. Next, we applied high-performance liquid chromatography (HPLC) analysis to a concentration series of *Dr*PSM variants. The dark-adapted individual heterodimer variants (*Dr*PSM_ER_ and *Dr*PSM_RE_) eluted corresponding to the *Dr*PSM_mon_ control in all measured concentrations (1–50 μM) indicating a monomeric state (Fig. 2, *B* and *C*). Once the heterodimer variants were mixed, the retention of the ER/RE pair shifted to earlier elution times resembling the dimeric *Dr*PSM_WT_ at the highest concentration, which indicates heterodimerization (Fig. 2, *B* and *C*). Although *Dr*PSM_EE_ eluted as a monomer, *Dr*PSM_RR_ was not applicable for HPLC analysis individually likely due to variant instability in combination with extreme experimental conditions (Fig. S3*A*). However, once *Dr*PSM_EE_ and *Dr*PSM_RR_ were mixed, the sample expressed retention peaks corresponding to dimers (Fig. S3*A*). This speaks for increased stability of *Dr*PSM_RR_ due to heterodimerization with *Dr*PSM_EE_.

When lowering the concentration of the heterodimeric mixtures, the elution time increased corresponding to a shift in equilibrium to monomeric species (Figs. 2*B*, S3). A similar shift was obtained after red light illumination albeit to a greater extent than in dark over the same concentration range, indicating a weakened dimer interface in the Pfr conformation (Fig. S3). Interestingly, this effect was also observed for *Dr*PSM_WT_ as it became increasingly monomeric at sub-micromolar concentrations after red light illumination, whereas the dark-adapted samples remained dimeric at the same concentrations (Fig. S3, *B* and *C*). This indicates that the PSM dimerization strength is reduced by red light, and that this applies to both homo- and heterodimer pairs.

**Figure 3:**
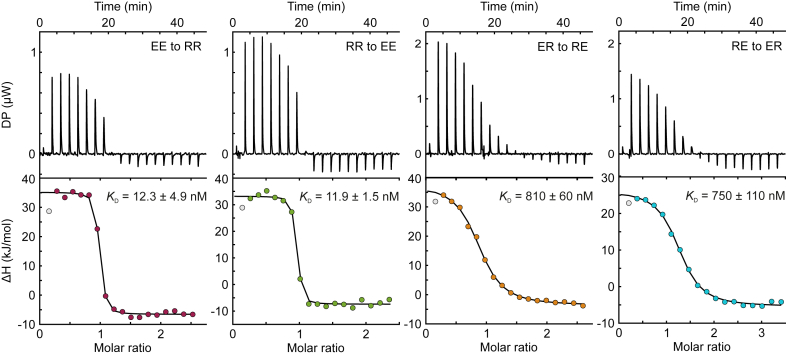
**Binding affinity of heterodimer variants determined with isothermal titration calorimetry (ITC).** There, differential power (DP) resulting from injections of a heterodimer variant to another is plotted against time, and the binding enthalpy (ΔH) is plotted against the molar ratio of these variants. See Fig. S4 for control titrations. All measurements were repeated at least three times with similar results. For concentration estimations, see Fig. S5.

To quantify the *Dr*PSM heterodimerization interactions, we applied isothermal titration calorimetry (ITC). There we titrated one subunit of the heterodimer variants with its cognate partner while detecting the heat of complex formation (Fig. 3). The positive signal indicates that the heterodimerization is an endothermic reaction, which is characteristic for formation of electrostatic interactions (36) and in line with the salt bridge modifications in the otherwise hydrophobic GAF–GAF interface of *Dr*BphP. Individual heterodimer or control variants did not result in a binding signal (Fig. S4), verifying that the endothermic signal in EE/RR and ER/RE titrations is caused by heterodimerization and not by component homodimerization. A brief initial rise of the signal was also observed (Fig. 3), which could have arisen from the hydrophobic interactions. Due to the unknown nature of its origin, this signal was not included in the analysis. The dissociation constant (*K*_D_) for the interaction between *Dr*PSM_ER_ and *Dr*PSM_RE_ was approximately 12 nM, whereas for *Dr*PSM_EE_ and *Dr*PSM_RR_ the *K*_D_ was approximately 800 nM at a 1:1 binding stoichiometry. By comparing these results to HPLC experiments (Fig. 2, *B*–*D* and Fig. S3) and molecular dynamics experiments done previously for *Dr*BphP (34), we can conclude that the affinities of ER/RE and EE/RR heterodimerization are weaker than the affinity of the *Dr*PSM_WT_ homodimerization.

### Heterodimeric PSMs can control HK output activity

Next, we investigated whether the heterodimeric phytochrome PSMs could be used to control enzymatic activity of HK OPMs. For this, we applied the heterodimer mutations to full-length *Dr*BphP (12) and to the artificial HK chimera *Dr*F1 (32, 37). *Dr*F1 is composed of *Dr*PSM and a HK module from *Bradyrhizobium japonicum* FixL (Fig. S1*A*) and acts as the underlying light-sensitive protein component in the optogenetic pREDusk tool (32) also applied later in this study. The wild-type variants (*Dr*BphP_WT_, *Dr*F1_WT_) are considered stable dimeric controls in the subsequent experiments. We also generated the monomeric control variants *Dr*BphP_mon_ and *Dr*F1_mon_ by introducing the monomerizing mutations F145S, L311E and L314E (33, 34).

The spectral properties of the individual *Dr*BphP and *Dr*F1 heterodimer variants (Fig. S6, *A* and *C*) resembled those of the corresponding *Dr*PSM variants (Fig. S2*A*). The dark reversion rates of the variant mixtures (EE/RR and ER/RE) did not correspond to those of their individual components (EE, RR, ER, RE) nor to an average of their cognate pairs (Fig. S6, *B* and *D*), suggesting interprotomer co-operativity. However, the extent of this co-operativity varied depending on the OPM. In HPLC analysis (Fig. S7), *Dr*BphP_EE_ eluted with a retention time corresponding to *Dr*BphP_mon_, whereas *Dr*BphP_RR_, *Dr*BphP_ER_, and *Dr*BphP_RE_ featured low-retention peaks corresponding to dimers like *Dr*BphP_WT_. The variant mixtures (*Dr*BphP_EE/RR_ and *Dr*BphP_ER/RE_) presented distinct dimer peaks, which indicates that all four individual variants favor heterodimerization over homodimerization. As for *Dr*F1, the retention of individual variants resembled that of *Dr*F1_mon_ indicating a monomeric form, whereas the variant mixtures appeared mainly dimeric like *Dr*F1_WT_. *Dr*F1_RR_ did not elute from the column properly and therefore cannot be interpreted reliably.

**Figure 4:**
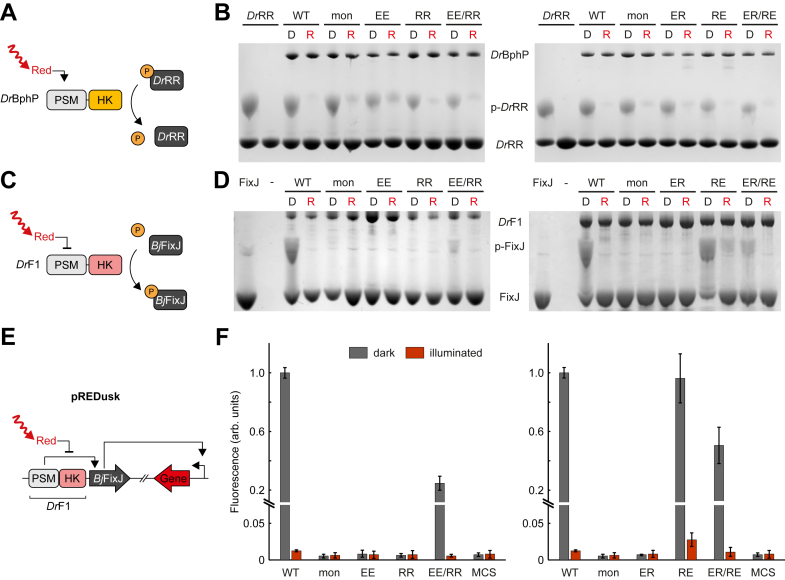
**Output activity of heterodimeric HK variants**. *A*, schematic presentation of the red light-induced dephosphorylation reaction catalyzed by *Dr*BphP. *B*, phosphatase activity of *Dr*BphP variants at 0.9 μM total concentration analyzed with Phos-tag assay. Each *Dr*BphP sample was supplemented with phosphorylated *Dr*RR (p-*Dr*RR) and incubated either in dark (D) or under red light (R). Phosphatase activity is seen from the reduced p-*Dr*RR amount. *C*, schematic presentation of the *red light*-repressed kinase activity of *Dr*F1. *D*, kinase activity of *Dr*F1variants at a total concentration of 0.9 μM (*left*) or 3.6 μM (*right*) analyzed with Phos-tag assay as in (B). The samples were supplemented with FixJ, and the accumulation of its phosphorylated form (p-FixJ) indicates net kinase activity. See Fig. S9*A* for equivalent experiments conducted at alternative concentrations. *E*, schematic presentation of the pREDusk tool adapted from (32). There, *Dr*F1 phosphorylates the FixJ response regulator in the dark, which leads to p-FixJ binding to a promoter region and subsequent expression of the target gene. *Red light* inhibits *Dr*F1 kinase activity and therefore target gene expression. *F*, *Ds*Red reporter expression by heterodimeric pREDusk tools in comparison to the original pREDusk tool (WT) (32), shown as mean ± SD of three biological repeats. The individual pREDusk variants shown here have kanamycin resistance, whereas EE and ER variants have streptomycin resistance in the EE/RR and ER/RE combinations. See Fig. S9*B* for tool variants with inverted resistances and alternative plasmid combinations, and Fig. S9*C* for bacterial plates expressing the pREDusk variants. All results were repeated at least three times with similar results.

The phosphatase activity of *Dr*BphP and its variants was studied *in vitro* with a Phos-tag assay that separates phosphorylated proteins from their unphosphorylated counterparts on a gel matrix (Fig. 4, *A* and *B*). We have previously revealed that *Dr*BphP functions exclusively as a phosphatase (12), and hypothesized here that *Dr*BphP dimerization is necessary for dephosphorylation of its cognate response regulator *Dr*RR (13). Thus, we expected overall phosphatase activity to be lost in the monomeric samples (*e.g.*, *Dr*BphP_mon_ and *Dr*BphP_EE_) and to be recovered only once the protomers form dimers (*Dr*BphP_WT_, *Dr*BphP_EE/RR,_ and *Dr*BphP_ER/RE_). All *Dr*BphP variants were incubated with phosphorylated *Dr*RR (p-*Dr*RR) either in dark or under saturating red light. Surprisingly, all of our constructs showed phosphatase activity as represented on Phos-Tag gels by the decreased amount of p-*Dr*RR (Fig. 4*B*). Not only the dimeric complexes (*Dr*BphP_WT_, *Dr*BphP_EE/RR_ and *Dr*BphP_ER/RE_) were able to dephosphorylate p-*Dr*RR under red light (*i.e.*, in Pfr), but also the monomeric construct *Dr*BphP_mon_ and all four isolated heterodimeric variants (*Dr*BphP_EE_, *Dr*BphP_RR_ *Dr*BphP_ER_, and *Dr*BphP_RE_) showed phosphatase activity. This implies that the phosphatase activity of *Dr*BphP is not dependent on GAF-GAF dimerization. In previous studies, we have shown that red light induces HK dissociation in *Dr*BphP_mon_ and *Dr*BphP_WT_ (8, 34), suggesting that phosphatase activity here would not depend on dimerization of HK modules either. To investigate whether interaction with *Dr*RR could affect *Dr*BphP dimerization, we conducted native polyacrylamide gel electrophoresis (PAGE) analysis at the same conditions as the activity assays (Fig. S8, *A* and *B*). Although *Dr*RR did not seem to induce dimerization, all *Dr*BphP variants contained dimeric species to some extent, which makes it difficult to estimate how the monomeric species contribute to phosphatase activity. We have previously shown that *Dr*BphP has some phosphatase activity also in the dark (12), which is apparent also here as a small decrease in p-*Dr*RR intensity relative to the *Dr*RR-only control (Figs. 4*B*, S9*A*). This phenomenon was even more pronounced when the concentration of individual *Dr*BphP heterodimer variants or their mixtures increased four-fold and the amount of p-*Dr*RR became the limiting factor (Fig. S9*A*).

We have previously shown that *Dr*F1 adopts net kinase activity in the Pr state (Fig. 4*C*) (32, 37). We therefore investigated using Phos-tag assay whether the heterodimer mutations affect this activity (Fig. 4*D*). For this, we incubated the *Dr*F1 variants in the presence of their cognate response regulator FixJ (32, 38). Kinase activity was then observed as an increased amount of phosphorylated FixJ (p-FixJ), which is clearly visible for *Dr*F1_WT_ in the dark (Fig. 4*D*). This effect is consistent with earlier results of chimeric photoreceptors bearing a FixL HK, which show that FixJ is phosphorylated in the dark (32, 37, 39, 40). When the sample contained only one type of heterodimer variant or *Dr*F1_mon_, no kinase activity was observed. The only exception was *Dr*F1_RE_ which showed red light-repressed kinase activity, thus indicating non-specific homodimer formation. The homodimerization of individual *Dr*F1 variants is not promoted by the presence of FixJ (Fig. S8, *C* and *D*), suggesting that the tendency of *Dr*F1_RE_ to homodimerize stems from the variant itself. As this homodimerization was not visible in native-PAGE, we deem it much weaker than that of *Dr*F1_WT_. Once the heterodimer variants were mixed with their counterparts, kinase activity was restored. When decreasing the *Dr*F1_ER/RE_ pair concentration to 1 μM, no kinase activity was detected, whereas the *Dr*F1_EE/RR_ pair maintained its kinase activity at this lowered concentration (Fig. S9*A*). This observation is consistent with the ITC results measured for the *Dr*PSM heterodimer pairs (Figs. 3, S4), indicating almost 10 times stronger affinity for the PSM_EE_/PSM_RR_ pair than for the PSM_ER_/PSM_RE_ pair.

We further expanded the kinase activity studies to living bacteria by utilizing the optogenetic gene expression tool pREDusk that encodes both *Dr*F1 and FixJ (32) (Fig. 4*E*). To visualize kinase activity, the pREDusk system was engineered to incorporate a reporter gene encoding for the red fluorescent protein *Ds*Red. Consequently, reporter gene expression represents FixJ phosphorylation by the FixL HK module of *Dr*F1. The heterodimerizing and monomerizing *Dr*PSM mutations were introduced into the pREDusk-*Ds*Red circuit and transformed into *Escherichia coli*. As a blank control, pREDusk with a multiple cloning site (MCS) instead of the *Ds*Red gene was used, as presented previously (32). The bacteria were first incubated either in darkness or under saturating red light, after which *Ds*Red expression was quantified from the fluorescence of the bacteria (Fig. 4*F*). These results agreed very well with the results observed using Phos-tag assays (Fig. 4*D*). With the exception of RE, the bacterial cells containing only one type of heterodimer variant or *Dr*F1_mon_ did not express *Ds*Red and resembled the negative MCS control. When both components of the heterodimer pairs were introduced into the cells, *Ds*Red fluorescence was restored. Furthermore, *Ds*Red expression by pREDusk variants was diminished under red light, which corresponds to halted net kinase activity of the underlying *Dr*F1 component. It is also worth noting that the residual expression seen under red light is completely absent in isolated heterodimer variants (excluding RE), indicating a complete absence of kinase activity. Taken together, these results showcase that the heterodimeric phytochrome PSMs can be used to control OPM activities both *in vitro* and in bacteria through a combination of light and oligomerization. Notably, the EE/RR variant pair shows more promise for end-use applications than the ER/RE pair, because neither *Dr*F1_EE_ nor *Dr*F1_RR_ individually produce a background signal.

## Discussion

In this study, we introduce modifications that enable the formation of stable phytochrome heterodimers on demand. In general, stable protein heterodimers can be engineered by introducing electrostatic interactions, like salt-bridges (41), or sterically complementary “knobs-into-holes” mutations (42) at the dimerization interface. We used the former approach by modifying two prominent salt bridges at the GAF-GAF dimerization interface. Our design yielded two *Dr*PSM heterodimer pairs, EE/RR and ER/RE, that dimerize with nanomolar affinity (12 nM and 800 nM, respectively) only once mixed (Figs 2, 3, S3, S4). The dimerization interface was slightly weakened by red light, an effect that was also detected for the *Dr*PSM_WT_ homodimer (Fig. S3). This observation is consistent with isolated PSMs of other phytochromes forming mixtures of monomers and dimers, such as Cph1 and Cph2 from *Synechocystis* PCC6803 (43, 44, 45, 46, 47) and Agp1 from *Agrobacterium fabrum* (48). While certain monomerizing mutations in the GAF-GAF interface have been introduced and presented previously (33, 34), this is the first time when both dimerization and monomerization of BphPs can be specifically controlled with certain interface mutations. The corresponding variants retain the photochromic properties characteristic for BphPs (Fig. S2) and enable the control of effector activities in response to light, as demonstrated here in the case of HK OPMs (Fig. 4). It is however worth mentioning that one PSM variant, RE, was able to form functional homodimers when fused to a HK module (Fig. 4, *D* and *F*). As HK modules provide a second dimerization interface in BphPs (8, 34), it is possible that the RE mutations in the PSM are not sufficient to counteract the overall dimerization. This problem, however, would not likely arise when combining PSMs with natively heterodimeric OPMs and is certainly absent in the EE/RR heterodimer pair.

Although a vast majority of analyzed BphPs function as homodimers (4, 7, 49), the formation of heterodimers has been observed for individual bacterial organisms expressing more than one phytochrome type. For example, two tandem bacteriophytochromes, *Rp*BphP2 and *Rp*BphP3, in *Rhodopseudomonas palustris* have been reported to form heterodimers once mixed *in vitro* (50). Agp1 and Agp2 are reported to interact in *A. fabrum*, although no protomer swapping has been observed (51). Plant type II phytochromes have also been shown to form heterodimers in *Arabidopsis thaliana* (52, 53), but their mode of dimerization differs notably from BphPs, as revealed by cryogenic electron microscopy (54). Therefore, the salt bridge-based modifications presented here for *Dr*BphP are not applicable to plant phytochromes.

**Figure 5:**
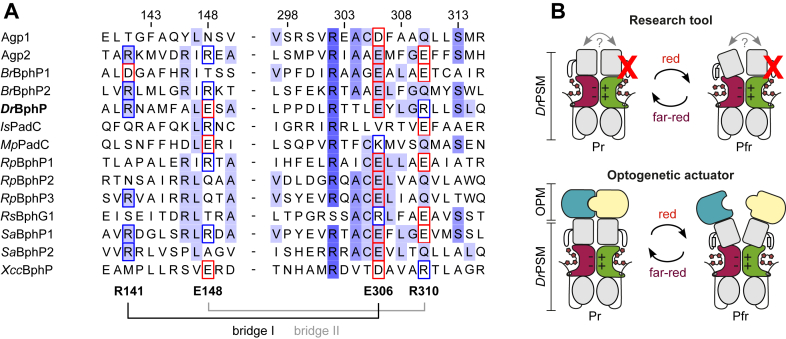
**Conservation of the GAF interface salt bridges and potential heterodimer tools**. *A*, representative BphP sequences from an extensive BphP sequence alignment. Residue numbering corresponds to the *Dr*BphP sequence. Residues involved in the salt bridges are indicated with a square colored according to their charge: *blue* = positive, *red* = negative. Residue conservation is indicated by hues of *blue* (≥84%, ≥ 68% ≥ 40%, < 40%), based on the Jalview coloring option “Percentage Identity” (72). Phytochrome sequences are as follows: Agp1 (UniProt Q7CY45) and Agp2 (UniProt A9CI81) from *A. fabrum*; *Br*BphP1 (UniProt Q8VUB6) and *Br*BphP2 (UniProt A4YPN9) from *Bradyrhizobium* sp. ORS 278; *Dr*BphP (UniProt Q9RZA4) from *D. radiodurans*; *Is*PadC (UniProt F7RW09) from *Idiomarina* sp. A28L; *Mp*PadC (UniProt A0A1I3XPG4) from *Marinobacter persicus*; *Rp*Bphp1 (UniProt A0A0D7EKU9), *Rp*BphP2 (UniProt Q6N5G3) and *Rp*BphP3 (UniProt Q6N5G2) from *Rhodopseudomonas palustris*; *Rs*BphG1 (NCBI WP_097082434) from *Rhodobacter* sp. JA431; *Sa*BphP1 (UniProt A0A1H8AQZ3) and *Sa*BphP2 (NCBI WP_002609371) from *Stigmatella aurantiaca;* and *Xcc*BphP (UniProt A0A0H2XCS3) from *Xanthomonas campestris*. For a sequence logo of the corresponding regions, see Fig. S10. *B*, schematic presentation of two potential uses for heterodimeric *Dr*PSM variants. First, interprotomer communication (denoted by “?”) can be studied by introducing asymmetric modifications (“X”) in the dimer. In this example, light-induced structural changes are limited to only one protomer. Second, heterodimeric output modules (OPMs) can be specifically controlled through light-induced conformational changes of the PSM heterodimers. This results in a heterodimeric optogenetic actuator, which can be, *e.g.*, a heterodimeric enzyme.

The resounding success of engineering artificial heterodimers through only two point mutations highlights the importance of the corresponding salt bridges for *Dr*BphP dimerization and raises the question of how well conserved these residues are across bacterial phytochromes—especially in light of how many BphPs have been shown to be functional dimers (4, 7, 49). According to a sequence analysis of 403 BphP species (Fig. 5*A*), these salt bridges are not strictly conserved, with “bridge I” being more conserved than “bridge II” (Fig. S10). In particular, the residues R141 and E148 (*Dr*BphP numbering) reside in an area of relatively low general conservation, although many BphPs still feature charged residues in the corresponding positions. For example, Agp1 or *Rs*BphG1 lack the capacity to form these two salt bridges, whereas Agp2 and *Sa*BphP1 could in theory form both. As the GAF interface constitutes one of the two typical major dimerization interfaces in BphPs (16), it is interesting that these stabilizing interactions are not strictly conserved across bacterial phytochromes. These salt bridges may thus provide an additional means for fine-tuning PSM dimerization and hence functional adaptation. The limited conservation also means that the heterodimerizing variants presented in this study may be applicable to only a subset of BphPs, therefore making our introduced *Dr*PSM modules the prime candidate for future applications.

One incentive to generate heterodimeric phytochrome modules arises from their potential uses as research tools or optogenetic actuators (Fig. 5*B*). To prove the concept, we applied the heterodimerizing mutations to two different SHKs, *Dr*BphP and the *Dr*F1 chimera. Although SHKs are a highly abundant type of sensor kinases (13, 55, 56), insight into the molecular mechanisms involved in HK activity remains limited, partially due to the relatively small amount of suitable molecular models that are soluble and comprise both sensor and OPMs (13). There is a wide consensus in the field of SHK research that kinase activity requires a dimeric OPM arrangement (13, 14, 15), with some exceptions among EL346-like light-oxygen-voltage (LOV) HKs (57, 58). The HK autophosphorylation reaction can occur either between sister protomers (*trans*) or within one protomer (*cis*), and the determinant for this lies in the DHp loop orientation (14, 15). Our results support the autophosphorylation reaction occurring in *trans* for the FixL HK, as the monomeric *Dr*F1 variants were incapable of phosphorylating FixJ (Fig. 4*D*). This *trans* phosphorylation is also supported by the crystal structures of YF1 and FixL (39, 59). In contrast, *Dr*BphP did not require GAF-GAF dimerization for light-controlled phosphatase activity (Fig. 4*B*). This suggests that the phosphatase activity of its HK module could lie within one protomer and occur in *cis*, which is also supported by our recent cryogenic electron microscopy model where the HK modules of *Dr*BphP separate upon red illumination (8). However, our native-PAGE analysis of monomeric *Dr*BphP variants also suggests the presence of dimeric species in the Phos-tag activity assay conditions (Fig. S8, *A* and *B*). Therefore, it cannot be fully determined at this time whether phosphatase activity arises from the monomeric or dimeric species, or both.

These insights into HK activity regulation in *Dr*BphP and *Dr*F1 highlight the applicability of the presented heterodimer variants for both BphP and SHK research (Fig. 5*B*). Due to the modular nature of *Dr*PSM and its ability to control HK modules from other sources (12, 32), the heterodimeric *Dr*PSM variants are not only a valuable tool for SHK studies, but can also be used to investigate interprotomer communication and function of other homo- and heterodimeric OPMs, like for example phosphodiesterases (21, 22), guanylate/adenylate cyclases (23, 24), di-guanylate cyclases (25, 26), or tyrosine kinases (27, 28). The ability to form specific heterodimer pairs on demand enables the generation of asymmetric modifications (exemplified as “X” in Fig. 5*B*), thus permitting investigation of the signal relay and integration processes between and within the protomers. Such asymmetric mutations have been previously applied to determine how the catalytic centers of EcoRV function independently of each other (60), what the mechanism of autophosphorylation is in *Rp*BphP2 and *Rp*BphP3 (50), and how the DHp loops affect the autophosphorylation mechanism of SHKs (39, 59). However, these example studies relied on random formation of heterodimeric complexes once mixed, without any controllable preference for formation of heterodimers over homodimers, and would therefore greatly benefit from tools such as our heterodimeric *Dr*PSM variants.

By applying our salt bridge exchanges to the optogenetic pREDusk tool (32), we further demonstrated that the heterodimeric *Dr*F1 chimeras are also functional as light-controlled HKs in living cells, highlighting the optogenetic potential of these heterodimeric PSMs (Fig. 4*F*). Analogous to our approach, complementary charges have been introduced to the homodimer interface of the *Neurospora crassa* Vivid photoreceptor to create negative and positive “Magnet” pairs that can be specifically heterodimerized with blue light (29). This system, however, is not photochromic and senses light of shorter wavelengths that are not as optimal for mammalian applications as red/far-red light (18, 19, 20). Moreover, it is restricted to applications that rely on transient light-induced dimerization, as are the majority of optogenetic systems published to date (30, 31). In contrast, our heterodimeric phytochrome modules respond to red/far-red light through their BV chromophore and can be switched on and off by light. The slow dark reversion of *Dr*PSM modules (Figs. S2*B*, S6, *B* and *D*) eliminates the need for continuous light input, thus reducing phototoxicity. Furthermore, our heterodimeric *Dr*PSMs form stable heterodimers that control effector activity through light-induced conformational changes. This expands the applicable OPM repertoire to comprise heterodimeric effector pairs and protein-protein interactions, such as transcription factors (61, 62, 63), enzymes (64, 65), and kinases (66). These tools can be generated without undesired homodimer by-products and controlled by a stable dimeric protein framework (Fig. 5*B*).

## Experimental procedures

### Cloning and DNA material

The full-length phytochrome from *D. radiodurans* strain R1 (*Dr*BphP) and its PSM fragment (*Dr*PSM) in a pET21b(+) plasmid (Novagen) were a kind gift from Prof. Richard Vierstra (6, 67). The construct *Dr*F1 in pET21b(+) is described elsewhere (37), as well as *Dr*RR (12) and monomer mutants of *Dr*BphP fragments (*Dr*BphP_mon_, *Dr*PSM_mon_) (34). The heterodimerizing mutations (R141E, E148R, E306R, and R310E) were introduced to *Dr*BphP, *Dr*PSM, and *Dr*F1 using the QuikChange Lightning Multi Site-Directed Mutagenesis Kit (Agilent Technologies). For the heterodimerization assays in bacteria, the pREDusk-*Ds*Red plasmid was applied with either a kanamycin (KanR) or a streptomycin (StrR) resistance (32). For multiple cloning sites (MCS)-containing parent versions, see Addgene IDs 188,970 (KanR) and 188,972 (StrR). See Fig. S1*B* for the list of all constructs used in this study.

### Protein expression and purification

Sample expression and purification were conducted in the same way as described in (12). The *Dr*PSM, *Dr*BphP, and *Dr*F1 variants were expressed in *E. coli* strain BL21 (DE3), either in LB or TB, overnight at 20 to 24 °C. The cells were lysed with EmulsiFlex, after which the His_6_-tagged proteins were purified with Ni-NTA affinity purification using HisTrap columns (GE Healthcare) and buffer (30 mM Tris/HCl, pH 8.0) with a 1 to 500 mM imidazole gradient. Alternatively, this purification step was performed in phosphate buffer (50 mM Na-phosphate, 150 mM NaCl, pH 8) for improved performance. To acquire holoprotein, at least 10× molar excess of biliverdin hydrochloride (Frontier Scientific) was added and incubated with the sample overnight on ice, either before or after affinity chromatography. The samples were then purified using size-exclusion chromatography (HiLoad 26/600 Superdex 200 pg, GE Healthcare) in buffer (30 mM Tris/HCl, pH 8.0). The purified samples were finally concentrated to 15 to 30 mg/ml, flash-frozen, and stored at −80 °C. *Dr*RR purification is described elsewhere (12), and purified FixJ was a kind gift from Prof. Andreas Möglich (University of Bayreuth).

### Absorption spectroscopy

The UV-vis spectra and dark reversion of the samples were measured with an Agilent Cary 8454 UV-Visible spectrophotometer (Agilent). The samples were diluted to 1.0 μM in buffer (30 mm Tris/HCl, 150 mM NaCl, pH 8.0) to obtain an approximate *A*_700_ value of 0.1. The Pr and Pfr states were populated by 3-min illumination with saturating 782-nm and 661-nm light (Roithner Lasertechnik GmbH), respectively, followed by immediate data acquisition. Spectra were baseline corrected at 850 nm and normalized to the 280 nm absorption. Dark reversion data were recorded at 15 min intervals after red illumination. All reversion measurements were performed in the dark at ambient conditions (room temperature). The two-component exponential fits for dark reversion data were calculated with Matlab R2023b (23.2.0.2459199) (MathWorks Inc.) using Eq (1):

(1) $$\frac{A_{750}}{A_{700}}\left(t\right)=A_{1}e^{-\frac{t}{\tau_{1}}}+A_{2}e^{-\frac{t}{\tau_{2}}}\text{,}$$

where *A*_700_ and *A*_750_ are absorption values, *t* is time (min), *A*_1_ and *A*_2_ are the decay amplitudes of the absorbance ratio, and *τ*_1_ and *τ*_2_ are the time constants of the decay component (min).

### Analytical size-exclusion chromatography

FPLC detection with Superdex-200 Increase 3.2/300 (GE Healthcare) was conducted in buffer (30 mm Tris/HCl, 150 mM NaCl, pH 8.0), and the absorption of proteins was detected at wavelengths 280 nm, 405 nm, 700 nm, and 750 nm. For each run, 24 μl of sample mixture (5 mg/ml or 90 μM total concentration) was eluted at a 50 μl/min flow rate. HPLC analysis with multiwavelength detection was conducted as previously described (12, 68). Experiments were executed with a bioZen 1.8 μm SEC-3 (150 mm × 4.6 mm) column (Phenomenex), buffer (30 mm Tris/HCl, 150 mM NaCl, pH 8.0) as a mobile phase, and at 350 μl/min flowrate using the LC-30AD Nexera Liquid chromatograph pumping system (Shimadzu Corporation) at 25 °C. In the case of full-length *Dr*BphP and *Dr*F1 variants, 10% acetonitrile was included in the mobile phase. The eluant was detected at 4.16667 Hz or 0.78125 Hz with a diode array UV-Vis detector (SPD-M20A, Shimadzu). For each run, 10 μl of sample mixture with 0.25 to 50 μM concentrations (depending on the experiment) was injected. For all SEC experiments, the Pr and Pfr states were populated by pre-illumination with 782 nm or 661 nm light (Roithner Lasertechnik GmbH), respectively. The retention volumes and times were estimated from the data with six-point Gaussian fits with Matlab R2023b (23.2.0.2459199) (MathWorks Inc.). The apparent molecular weights were determined with calibration curves calculated based on the elution of a Bio-Rad protein standard containing marker proteins Vitamin B12 (1.35 kDa), myoglobin (17 kDa), ovalbumin (44 kDa), γ-globulin (158 kDa), and thyroglobulin (670 kDa), and Eq. 2:

(2) $$K_{av}=\frac{V_{e}-V_{0}}{V_{t}-V_{0}}\text{,}$$

where *K*_av_ is the distribution coefficient, *V*_e_ is the elution volume, *V*_0_ is the void volume, and *V*_t_ is the total volume of the column. See Fig. S3, *D* and *E* for representative standard chromatograms and calibration curves. To account for the small changes in retention between measurement days, the retentions of presented chromatograms were adjusted to the nearest maxima in the molecular weight standard.

### Isothermal titration calorimetry

Isothermal calorimetry was conducted with MicroCal PEAQ-ITC (Mavern Pananalytical), as previously described (12). The effect of biliverdin incorporation was considered in concentration determination by multiplying the *A*_280_ value by 0.84, as specified in Fig. S5. For the titration, the sample cell was loaded with 9 to 18 μM (300 μl) of protein in buffer (30 mM Tris/HCl, 150 mM NaCl, pH 8.0), and the injection syringe was loaded with 100 to 280 μM (75 μl) of interacting protein. To populate the Pr state, the samples were briefly pre-illuminated with 782 nm light (Roithner Lasertechnik GmbH). The measurements were conducted with 750 rpm stirring at 25 °C and in darkness. The injection scheme started with a 0.4 μl sample injection, followed by 2-μl injections every 150 s. The background signal for individual EE, RR, ER and RE components was estimated by injecting protein into buffer and *vice versa*. All data from triplicate experiments were analyzed using the ORIGIN 7-based MicroCal PEAQ-ITC Analysis Software version 1.41 (Malvern Panalytical). The curves were fitted into a single-site binding isotherm with the first injection excluded. The *K*_D_ values were reported as ±SD from three repeats.

### *In vitro* activity assays

Phos-Tag detection was conducted in the same way as described in Multamäki *et al.* (2021) (12). To generate phosphorylated *Dr*RR (p-*Dr*RR), protein was incubated for 30 min at 37 °C with 90 mM acetyl phosphate in buffer (25 mM Tris/HCl pH 7.8, 5 mM MgCl_2_, 4 mM 2-mercaptoethanol, 5% ethylene glycol) following buffer exchange to (30 mM Tris/HCl, pH 8.0). The phosphatase or kinase reactions with either 16 μM p-*Dr*RR or 13 μM FixJ, respectively, and desired phytochromes (at 0.9 μM or 3.6 μM total concentration) were conducted in buffer (25 mM Tris/HCl pH 7.8, 5 mM MgCl_2_, 4 mM 2-mercaptoethanol, 5% ethylene glycol) with a total volume of 10 μl. The reactions were initiated with 2 mM ATP and continued with incubation (25 °C) in dark or under saturating 657-nm red light for 10 min for phosphatase reactions and for 20 min for kinase reactions. For the mobility shift detection of phosphorylated *Dr*RR or FixJ, we applied Zn_2+_-Phos-tag SDS-PAGE assay (Wako Chemicals), where samples were run through 9% SDS-PAGE gels containing 2.5 μM Phos-tag acrylamide at 40 mA/gel, according to manufacturer instructions.

### Activity assays in bacteria

For the heterodimerization assays in bacteria, the pREDusk-*Ds*Red plasmid variants with kanamycin and streptomycin resistance were used (see Fig. S1*B*). The pREDusk variants “WT” and “mon” were used as controls, as they should either form stable homodimers (WT) or not dimerize at all in physiological concentrations (mon). pREDusk-MCS was used as a bacterial control. The plasmids with either kanamycin resistance, streptomycin resistance, or both were transformed into *E. coli* DH5α cells, plated on LB agar plates with the relevant antibiotics (25 μg/ml kanamycin, 50 μg/ml streptomycin, or both), and incubated overnight in dark at 37 °C. The following day, the bacteria from the resulting colonies were streaked out on new LB-agar plates and incubated for 20 h either in dark or under red LED light (657 nm, ≤40 μW/cm^2^, Mightex). Small fragments from each sample were transferred to 100 μl of dH_2_O in three replicates and mixed, and their *A*_600_ and *Ds*Red fluorescence intensities [ex. (554 ± 5) nm; em. (591 ± 10) nm] were measured with a Tecan Spark microplate reader and the Spark Control V 3.1. SPI software. The results were normalized to the approximate amount of bacteria by dividing the fluorescence intensity (F_591_) by the absorbance at 600 nm (*A*_600_), and all data were normalized to the fluorescence of the kanamycin-resistant WT sample in dark. The experiment was repeated three separate times with three technical replicates, and the data presents the average ± SD from the repeats.

### Bioinformatics

The AlphaFold models (Fig. 1*C*) were generated in the ColabFold server (69) using AlphaFold2-multimer (70) with *Dr*BphP residues 1 to 321 as an input sequence. For sequence analysis (Fig. 5*A*), 41 previously published BphP sequences were added to 363 InterPro entries with a PAS (IPR013654) – GAF (IPR003018) – PHY (IPR013515) architecture, a full list is provided as Supplementary material. Sequences were then aligned using ClustalO (71) with default parameters through Jalview (72) and manually curated. The aligned sequence file is also provided as Supplementary material.

## Data availability

All data presented in the study are shared upon request from the corresponding author (heikki.p.takala@jyu.fi). These include the AlphaFold structure predictions (Fig. 1*C*); absorption spectra and dark reversions (Fig. 1*D* and *E*, Fig. S2, Figs. S5, and S6), chromatography data (Figure 2, Figs. S3, and S7), ITC data (Figs. 3 and S4), gels (Fig. 4*B* and *D*, Figs. S8, and S9*A*), activity assays (Figs. 4*F*, S9*B*), and bacterial plate images (Fig. S9*C*). Files underlying the sequence alignment (Fig. 5*A*) and frequence logo (Fig. S10) are provided as separate supplementary files.

## Supporting information

This article contains supporting information (12, 32, 34, 37, 73).

## Conflict of interest

The authors declare that they have no conflicts of interest with the contents of this article.

## Supporting information

Supporting information

Supp_sequences_2

Supp_sequences_aligned_2
