# Supplementary material for: Engineered bacteriophytochrome heterodimers for research and applications
Source: J Biol Chem. 2025 Jul 4;301(8):110452. doi: 10.1016/j.jbc.2025.110452 (PMC12329595; doi:10.1016/j.jbc.2025.110452)
Supplement: Supporting information [file mmc1.pdf]

Supporting Information for:

Engineered bacteriophytochrome heterodimers for research and applications

Iida Tuure[1], Cornelia Böhm[1], Jessica Rumfeldt[1], Elina Multamäki[2], and Heikki Takala[1,2,*]

[1] Department of Biological and Environmental Science, Nanoscience Center, University of Jyvaskyla, 40014 Jyvaskyla, Finland; [2] Department of Anatomy, University of Helsinki, 00014 Helsinki, Finland

* For correspondence: heikki.p.takala@jyu.fi, +358 46 923 6211 (H.T.)

This PDF file includes:

Figures S1 to S10
SI References

A

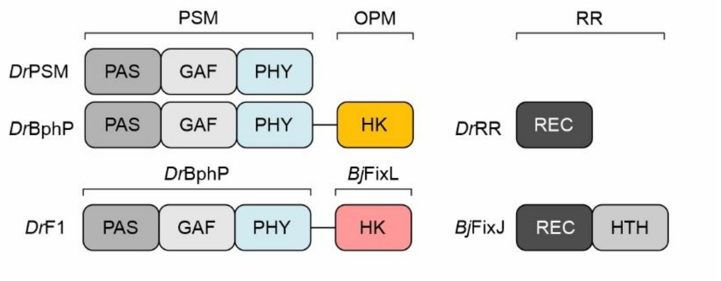

B

Short name	*Dr*PSM mutation	Parent plasmid	Reference
WT	-	pET21b(+)	(1−3)
Mon	F145S, L311E, L314E	pET21b(+)	(4), this work
ER	R141E, E148R	pET21b(+)	this work
RE	E306R, R310E	pET21b(+)	this work
EE	R141E, R310E	pET21b(+)	this work
RR	E148R, E306R	pET21b(+)	this work
MCS	-	pREDusk-MCS	(1−3)
WT	-	pREDusk-*Ds*Red	(1−3)
Mon	F145S, L311E, L314E	pREDusk-*Ds*Red	this work
ER	R141E, E148R	pREDusk-*Ds*Red	this work
RE	E306R, R310E	pREDusk-*Ds*Red	this work
EE	R141E, R310E	pREDusk-*Ds*Red	this work
RR	E148R, E306R	pREDusk-*Ds*Red	this work

Figure S1. Constructs used in this study. *A*, schematic presentation of the different constructs used in this study with their domain compositions. Protein definitions: *Dr*BphP (phytochrome from *Deinococcus radiodurans*), *Dr*PSM (photosensory module of *Dr*BphP), *Dr*F1 (chimera of *Dr*PSM and the *Bradyrhizobium japonicum* FixL histidine kinase module), *Dr*RR (response regulator from *D. radiodurans*), *Bj*FixJ (response regulator from *B. japonicum*). Domain abbreviations: PSM (photosensory module), OPM (output module), RR (response regulator), PAS (period/ARNT/single-minded), GAF (cGMP phosphodiesterase/adenylyl cyclase/FhlA), PHY (phytochrome-specific), HK (histidine kinase), REC (receiver), HTH (helix-turn-helix). *B*, DNA constructs and their abbreviations used in this study (1–4).

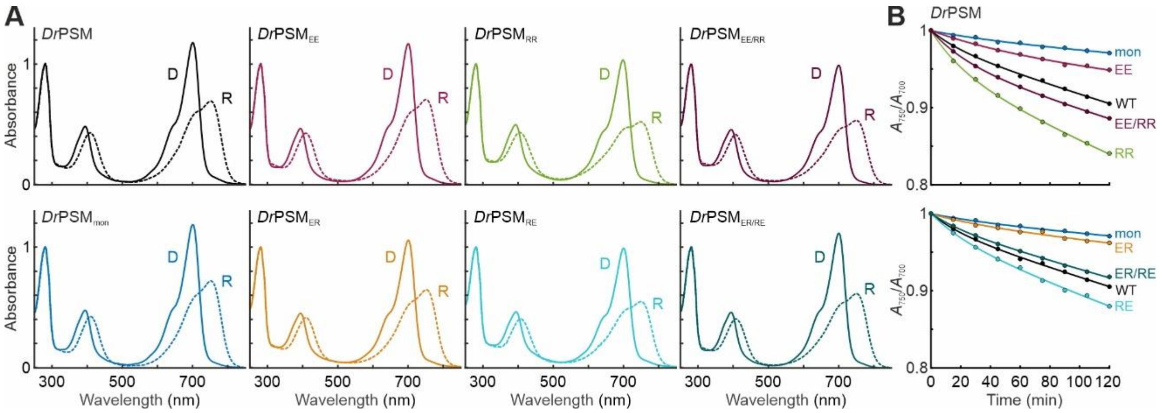

Figure S2. Absorption spectroscopy analysis of the *Dr*PSM variants. *A*, absorbance spectra of *Dr*PSM variants and heterodimeric mixtures in dark (D) and under red light (R). The spectra are normalized to the absorbance value at 280 nm. *B*, dark reversion of the *Dr*PSM variants and heterodimeric mixtures illustrated as decrease of the A_{750}/A_{700} ratio. The reversions of the mixtures (EE/RR and ER/RE) qualitatively resemble the reversion of the wild-type *Dr*PSM (WT). All reversions are normalized to the A_{750}/A_{700} ratio at zero timepoint.

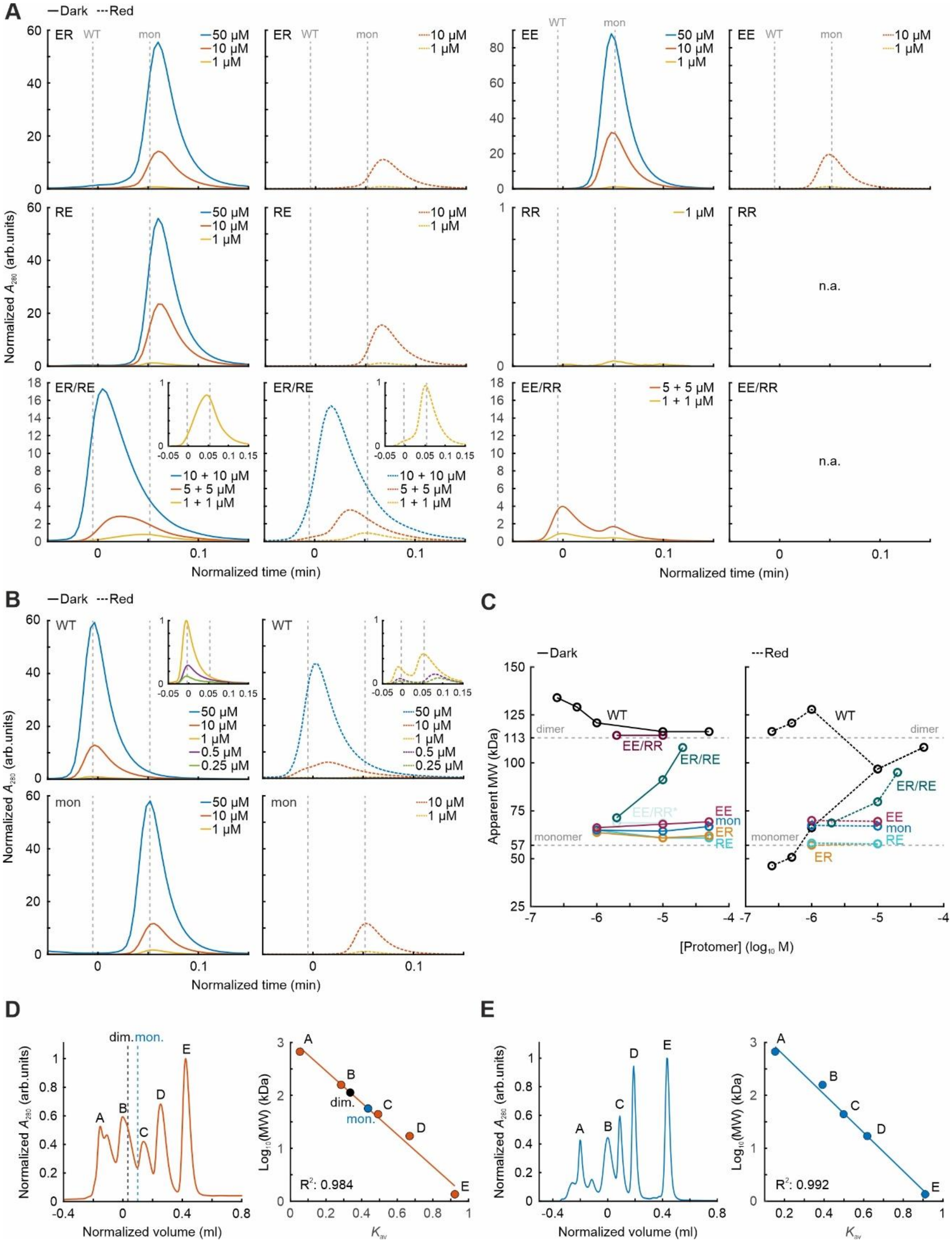

Figure S3. Chromatographic analysis of *Dr*PSM variants and heterodimer mixtures at varying concentrations and under different light conditions. *A*, HPLC retention plots of the *Dr*PSM variants in dark (solid lines) and after red light (dashed lines). Individual samples appeared monomeric, whereas variant mixtures indicated formation of heterodimers regardless of the illumination

status. *Dr*PSM$_{RR}$ did not elute from the column by itself, and only to a certain extend once mixed with *Dr*PSM$_{EE}$. These samples (*Dr*PSM$_{RR}$ and *Dr*PSM$_{EE/RR}$) could not be measured after red illumination. The vertical lines indicate the retention of dark-adapted *Dr*PSM$_{WT}$ (WT) and *Dr*PSM$_{mon}$ (mon), which are shown in panel *B*. *B*, HPLC of *Dr*PSM$_{WT}$ and *Dr*PSM$_{mon}$ control samples. The chromatograms are normalized to the dark-adapted *Dr*PSM$_{WT}$ at 1 µM concentration. The lowest concentrations are plotted in the inset for clarity. *C*, apparent molecular weights of dark-adapted and red illuminated *Dr*PSM variants plotted against increasing protein concentration. The monomeric fraction of the EE/RR mixture in dark (indicated with *) is likely excess EE protomers left due to the loss of RR. Theoretical molecular weights of the *Dr*PSM dimer (113 kDa) and monomer (57 kDa) are indicated as grey horizontal dashed lines. *D–E*, representative retention profiles of molecular weight standard proteins and calibration curves of FPLC (*D*) and HPLC (*E*) analyses. The peaks correspond to the retention of thyroglobulin (A), γ-globulin (B), ovalbumin (C), myoglobin (D) and vitamin B12 (E). For FPLC analysis, distribution coefficients (K_{av}) were calculated for theoretical the *Dr*PSM dimer (113 kDa, black) and monomer (57 kDa, blue) to illustrate theoretical retention of dimeric (dim.) and monomeric (mon.) species of *Dr*PSM in Fig. 2.

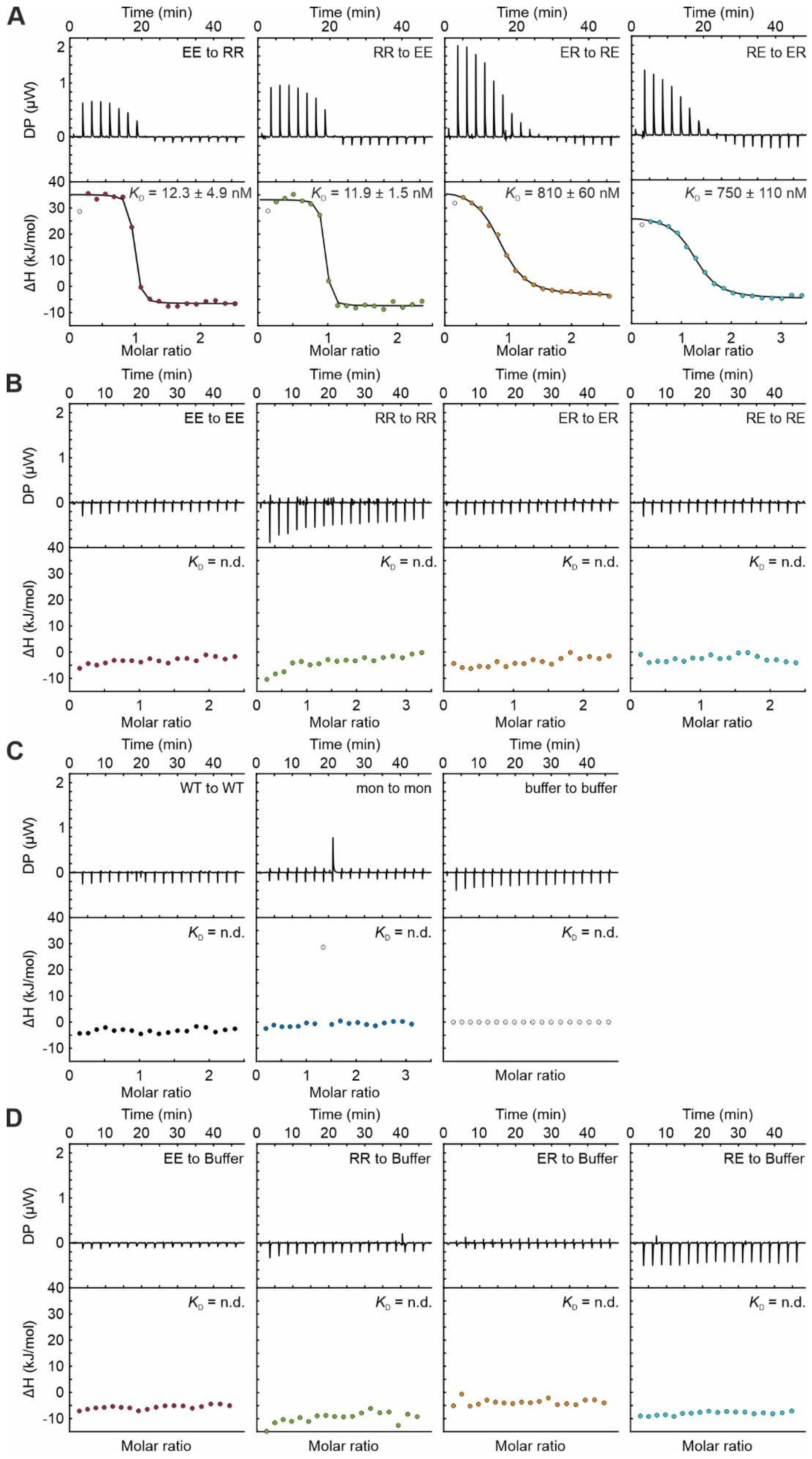
A
B
C
D
Time (min)
DP (µW)
ΔH (kJ/mol)
Molar ratio
EE to RR
RR to EE
ER to RE
RE to ER
K_D = 12.3 ± 4.9 nM
K_D = 11.9 ± 1.5 nM
K_D = 810 ± 60 nM
K_D = 750 ± 110 nM
EE to EE
RR to RR
ER to ER
RE to RE
K_D = n.d.
WT to WT
mon to mon
buffer to buffer
EE to Buffer
RR to Buffer
ER to Buffer
RE to Buffer

Figure S4. Isothermal titration calorimetry (ITC) of *Dr*PSM variant heterodimerization. *A*, titration between EE/RR and ER/RE heterodimer pairs. *B*, titration of heterodimer variants (EE, RR, ER, RE) to themselves. *C*, titration of wild-type (WT) and monomer mutant (mon) to themselves, and buffer to buffer. *D*, titration of heterodimer variants (EE, RR, ER, RE) to buffer. The K_D values of cognate heterodimer pairs agree well with each other, with heterodimerization values being (12.3 ± 4.9) nM and (11.9 ± 1.5) nM between EE and RR, and (810 ± 60) nM and (750 ± 110) nM between ER and RE. All heterodimerization reactions were endothermic with a positive ΔH signal. Control titrations and variant titrations to themselves produced small featureless negative signals, indicating that the sample measurements were minimally affected by homodimerization or secondary dilution/machine effects. All sample measurements (panel *A*) were repeated at least three times, and all control measurements (panels *B*–*D*) at least two times. DP = differential power, ΔH = binding enthalpy.

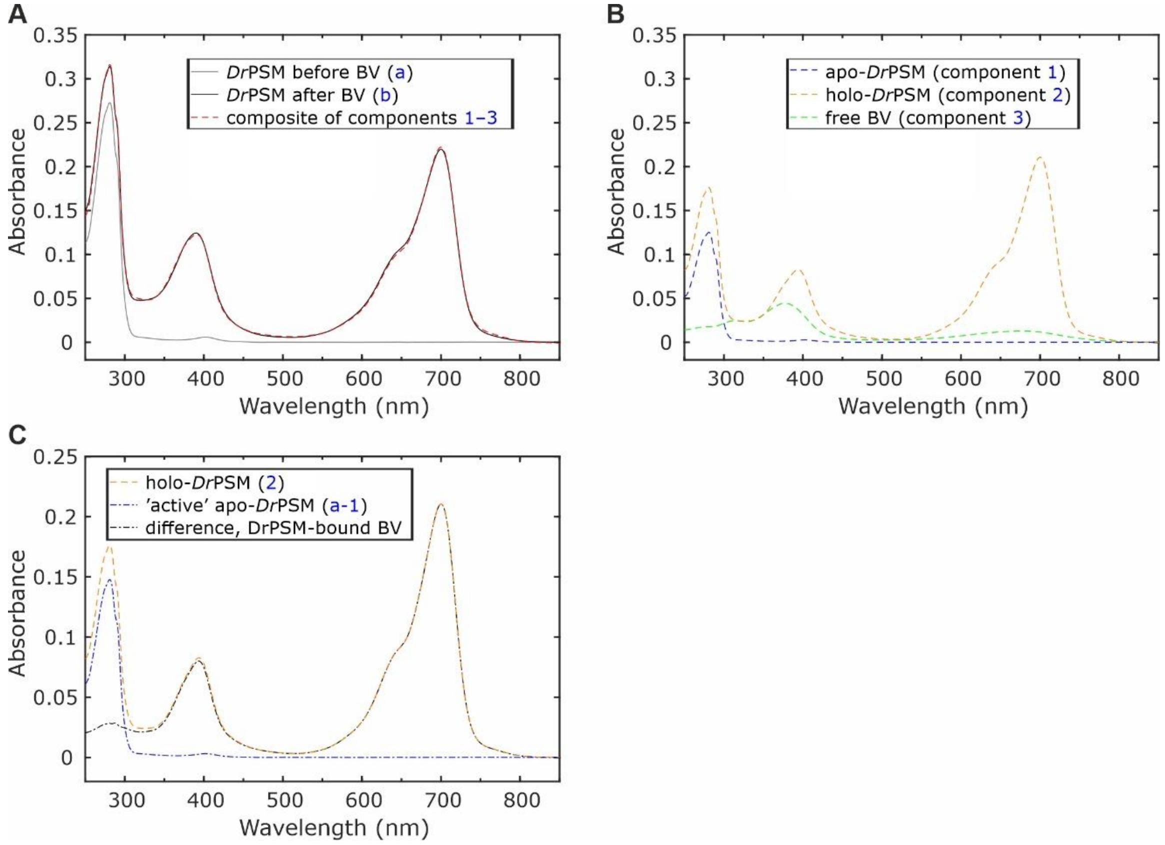

Figure S5. Determination of the biliverdin (BV) contribution to the 280-nm absorbance in BV-bound ('holo') *Dr*PSM. *A*, the absorbance spectra of BV-absent ('apo') *Dr*PSM at 3.7 µM monomer concentration, before (*a*, solid grey line —) and after (*b*, solid black line —) the addition of 3 µM BV. The red dashed line (- -) is the linear combination of the three absorbance spectra scaled as shown in panel *B*. *B*, the absorbance spectra of free BV (component 1, green dashed line - -), apo-*Dr*PSM (component 2, blue dashed line - -), and holo-*Dr*PSM (component 3, orange dashed line - -). The composition of the three components indicates that only a portion of apo-*Dr*PSM, referred to here as the 'active' portion, binds BV to form holo-*Dr*PSM (component 2), and that a 'non-active' portion remains in solution (component 1). Some added BV also remains in solution (component 3) even though it is not in excess. *C*, the 'active' apo-*Dr*PSM portion (blue dotted dashed line · -) was generated by subtracting the spectrum of the non-active portion of apo-*Dr*PSM (component 1) from the initial apo-*Dr*PSM spectrum ('*a*' in panel *A*). Subtracting the 'active' apo-*Dr*PSM spectrum from holo-*Dr*PSM (component 2) gives the difference spectrum of holo- *Dr*PSM vs. apo-*Dr*PSM and therefore represents the absorbance attributed to bound BV in holo-*Dr*PSM (dotted dashed black line · -). When calculating the protein concentration of holo-*Dr*PSM, the A_{280} was first multiplied by 0.84 which is the ratio of A_{280} values for the holo-*Dr*PSM vs. 'active' apo *Dr*PSM spectra shown in panel *C*. Alternatively, the value that bound BV contributes to the absorbance at 280 nm can be estimated to be equal to the absorbance of holo-*Dr*PSM at 350 nm. All spectra were measured in buffer (30 mM Tris/HCl, pH 8) with Agilent Cary 8454 UV-Visible spectrophotometer as described in the main text.

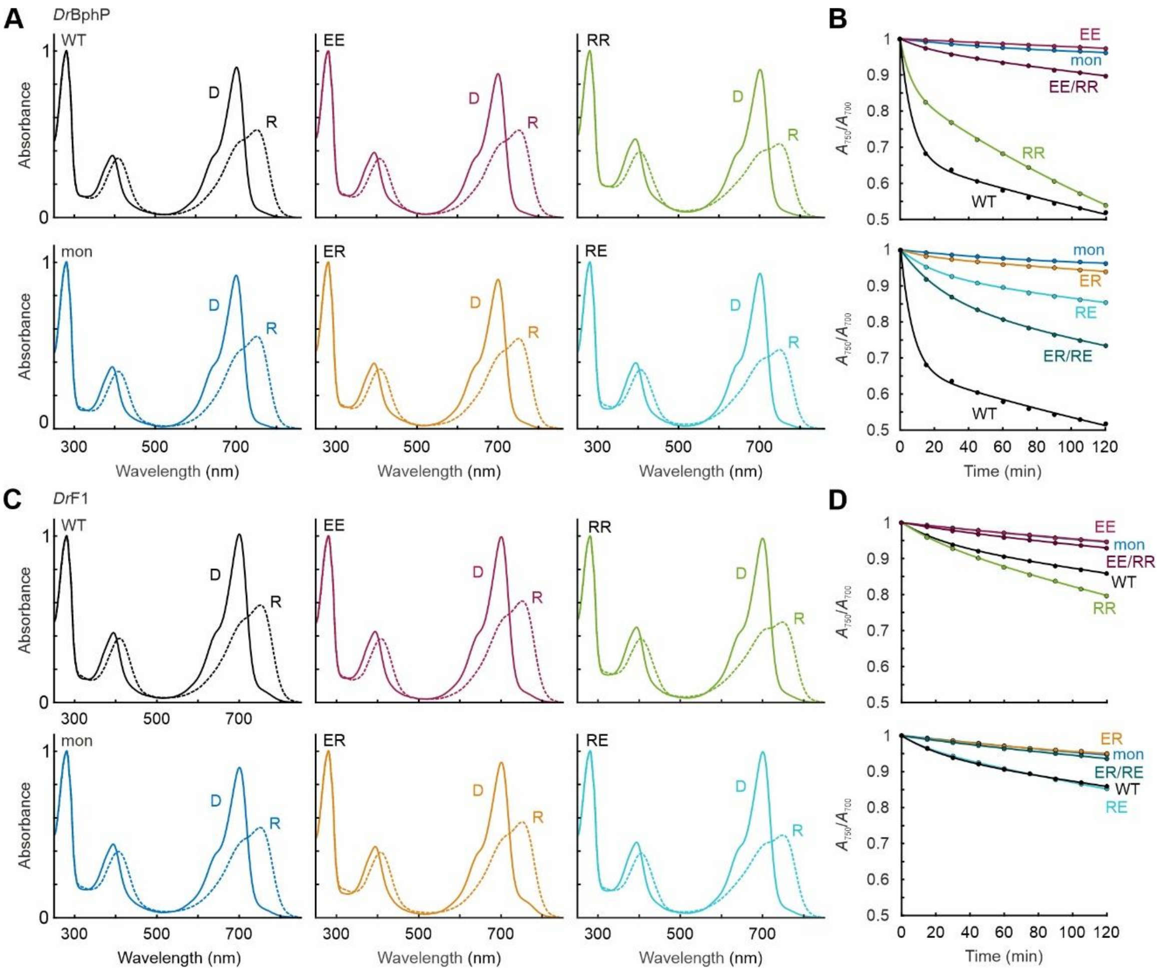

Figure S6. Absorbance spectra and dark reversion of *Dr*BphP (*A*–*B*) and *Dr*F1 (*C*–*D*) variants. The variant spectra resemble typical phytochrome-like spectra similar to the wild type *Dr*BphP (WT) both in dark (D) and under red light (R). The spectra are normalized to the 280 nm absorbance, and the reversions are normalized to the A_{750}/A_{700} ratio at zero timepoint. The dark reversions suggest interprotomer communication as the reversions of the variant mixtures are not direct averages of the reversions of the individual components. Notably, the reversion of *Dr*BphP$_{ER/RE}$ was faster than *Dr*BphP$_{ER}$ or *Dr*BphP$_{RE}$ and started to resemble the reversion of *Dr*BphP$_{WT}$.

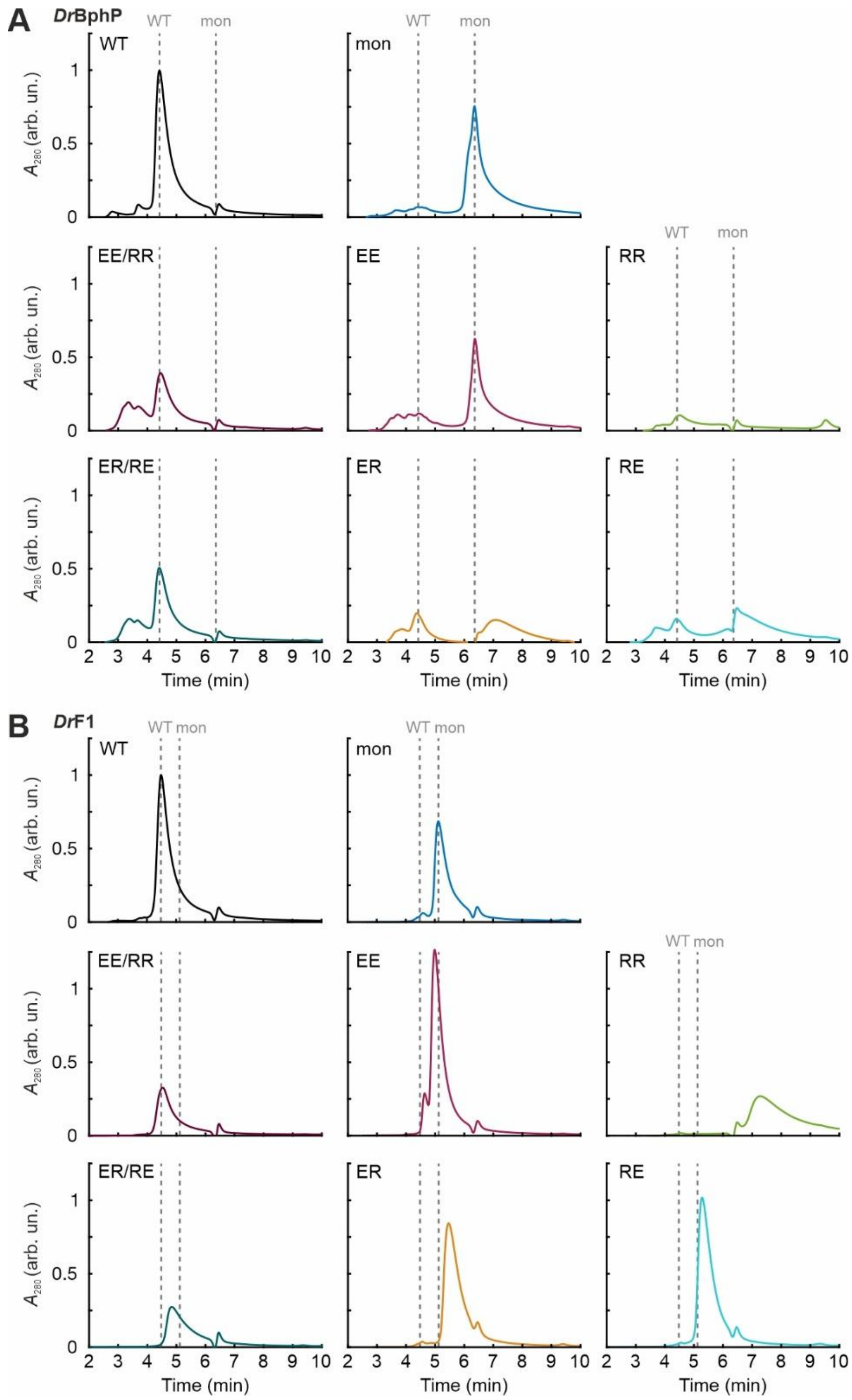

Figure S7. High performance liquid chromatography (HPLC) analysis of *Dr*BphP (*A*) and *Dr*F1 (*B*) variants as well as their heterodimer mixtures in dark. The heterodimer variant mixtures express larger dimer proportion compared to the individual variants. The vertical lines indicate the elution of the wild type (WT) and monomer (mon) constructs, and the chromatograms are normalized to the highest A_{280} value of the corresponding WT sample. Some variants (especially *Dr*BphP$_{RR}$ and *Dr*F1$_{RR}$) performed poorly in the HPLC, which is likely due to their incompatibility with the harsh experimental conditions.

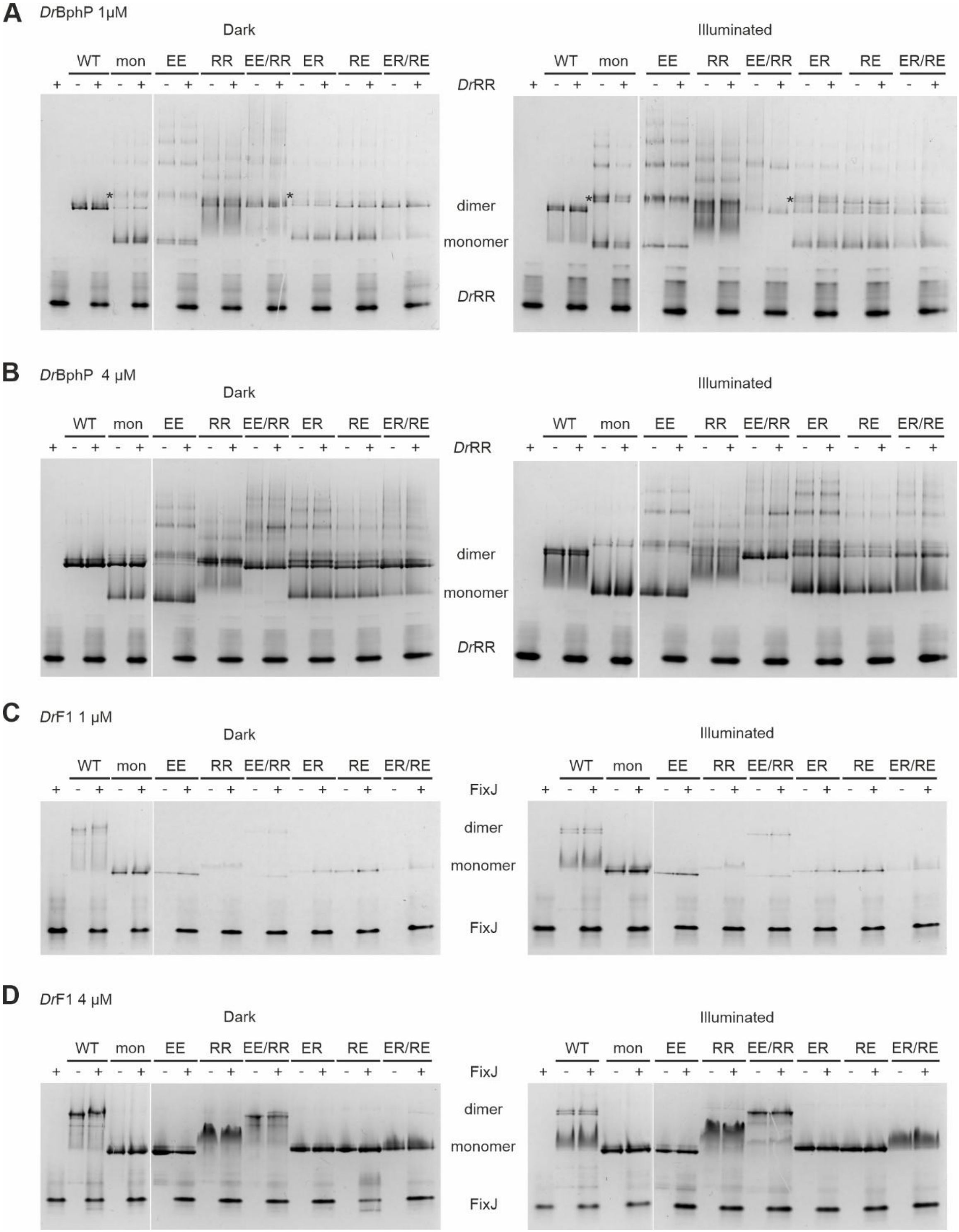

Figure S8. Native polyacrylamide gel electrophoresis (PAGE) gels of *Dr*BphP and *Dr*F1 variants with and without their cognate response regulators *Dr*RR and FixJ, respectively. Addition of the response regulator to each sample does not seem to affect its oligomeric state. All reactions were performed under conditions equivalent to the Phos-tag assays: 1 or 4 µM of *Dr*BphP or *Dr*F1 supplemented with 16 µM p-*Dr*RR or FixJ and 2 mM ATP in (25 mM Tris/HCl pH 7.8, 5 mM $MgCl_2$,

4 mM 2-mercaptoethanol, 5% ethylene glycol). The ‘Dark’ and ‘Illuminated’ samples were pre-illuminated with 782 nm or 661 nm light (Roithner Lasertechnik GmbH), respectively, before application to 4–16% gradient NativePAGE™ Bis-Tris Mini Protein Gels (Invitrogen). The gels were run at 150V for 1.5 hours on ice, either in darkness or under constant saturating red light (661 nm). All results were repeated at least three times. *A–B*. Among the *Dr*BphP variants, wild-type (WT) and heterodimer mixtures (EE/RR, ER/RE) appear mainly dimeric, and red light slightly monomerizes WT as well as the ER/RE pair, but not the EE/RR pair. RR variant appears as a monomer/dimer mixture partially monomerized by light. The variants mon, EE, ER and RE individually appear predominantly monomeric but also contain dimeric species. Interestingly, these variants feature an additional dimeric band that appears slightly higher than the WT band, especially after red light formation (marked as * in panel *A*). This additional band suggests the presence of dimeric species with an alternative dimerization scheme. *C–D*. Among the *Dr*F1 variants, the WT sample and both heterodimer mixtures (EE/RR and ER/RE) appear as mixtures of dimers and monomers, whereas the monomer variant (mon) and individual heterodimer variants (EE, RR, ER and RE) appear monomeric. Red light appears to monomerize the WT and the ER/RE pair, but the EE/RR pair seems almost unaffected.

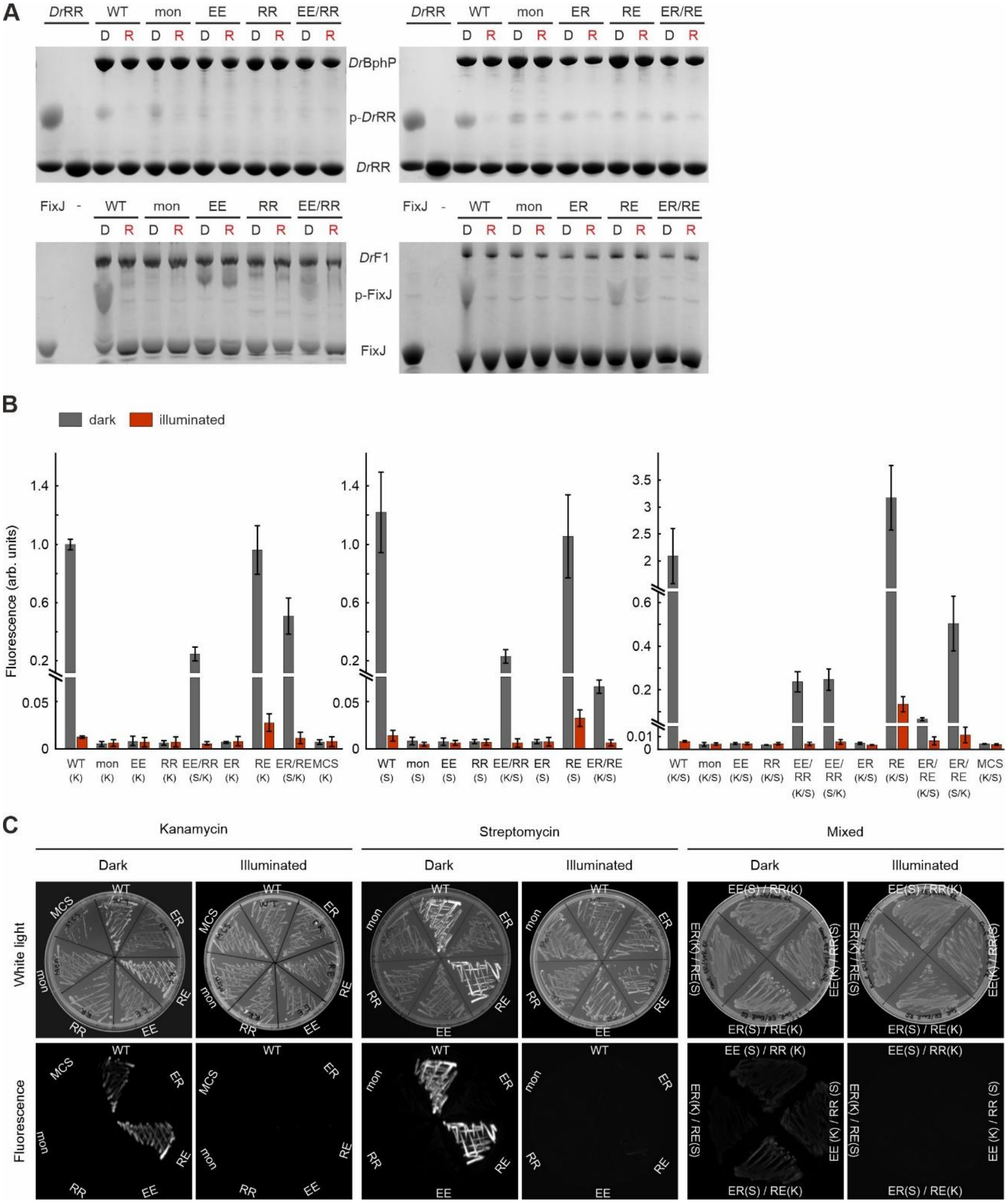

Figure S9. Control of HK output activity with the heterodimer variants. *A*, Phos-tag assays of *Dr*BphP and *Dr*F1 variants conducted at alternative concentrations to the main text Fig. 4. The phosphatase activity of *Dr*BphP variants at higher concentration (3.6 µM total concentration, upper gels) than in Fig. 4*B* (0.9 µM) increased to the point where red light control was masked in all of the samples except for *Dr*BphP$_{WT}$. Increased *Dr*F1 variant concentrations used in the lower left gel (3.6 µM compared to 0.9 µM in Fig. 4*D*) led to increased net kinase activity, but when lowering the concentrations in the lower right gel (0.9 µM compared to 3.6 µM in Fig. 4*D*), the kinase activity of *Dr*F1$_{ER/RE}$ diminished to undetectable. *B*, Bacterial pREDusk experiments shown

in Fig. 4*F*, their repeats with inverted kanamycin (K) or streptomycin (S) resistances, and experiments where all samples contain both K- and S-resistant plasmids. The strong expression levels of the WT and the RE variant in double-resistant (K/S) sample arrays can be explained by an increased probability of homodimerization over heterodimerization and by the combined effect of two origins of replication (ColE in K-plasmid and CloDF13 in R-plasmid). *C*, Bacterial plates expressing pREDusk variants and their combinations in dark or under red light. The plates were detected either with Trans-UV setting (λ = 302 nm, upper row) or with Epi-green setting with (520–545) nm excitation and (602 ± 25) nm emission to detect *Ds*Red reporter fluorescence (lower row). The results qualitatively agree with the experiments presented in Fig. 4*F* of the main text and the panel *B* of this figure.

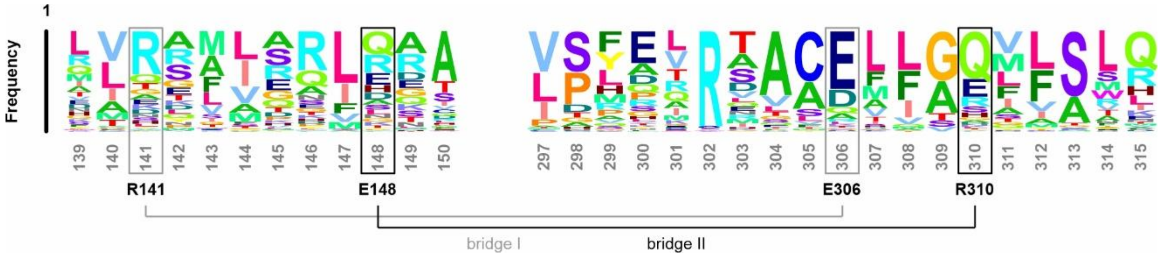

Figure S10. Frequency logo (residues are scaled relative to frequencies at each position) of 403 BphP sequences. Sequence logos do not allow empty positions, thus the ClustalO-aligned sequences were limited to the areas of interest. The logo was created using the *k*pLogo online tool (5). Numbering corresponds to *Dr*BphP residues. Among BphPs, the residues corresponding to bridge I (R141 and E306) are moderately conserved. Both residues involved in bridge II (E148 and R310) lack apparent conservation across bacteriophytochromes. In general, both interface regions containing the residues involved in forming these salt bridges show relatively low sequence conservation.

SI References

1. Multamäki, E., Nanekar, R., Morozov, D., Lievonen, T., Golonka, D., Wahlgren, W. Y., Stucki-Buchli, B., Rossi, J., Hytönen, V. P., Westenhoff, S., Ihalainen, J. A., Möglich, A., and Takala, H. (2021) Comparative analysis of two paradigm bacteriophytochromes reveals opposite functionalities in two-component signaling. *Nat. Commun*. **12**, 4394

2. Multamäki, E., García de Fuentes, A., Sieryi, O., Bykov, A., Gerken, U., Ranzani, A. T., Köhler, J., Meglinski, I., Möglich, A., and Takala, H. (2022) Optogenetic Control of Bacterial Expression by Red Light. *ACS Synth. Biol.* **11**, 3354–3367

3. Meier, S. S. M., Multamäki, E., Ranzani, A. T., Takala, H., and Möglich, A. (2024) Leveraging the histidine kinase-phosphatase duality to sculpt two-component signaling. *Nat. Commun*. **15**, 4876

4. Takala, H., Björling, A., Linna, M., Westenhoff, S., and Ihalainen, J. A. (2015) Light-induced Changes in the Dimerization Interface of Bacteriophytochromes. *J. Biol. Chem*. **290**, 16383–16392

5. Wu, X., and Bartel, D. P. (2017) kpLogo: positional k-mer analysis reveals hidden specificity in biological sequences. *Nucleic Acids Res*. **45**, W534–W538
